# Religiosity and Meditation Practice: Exploring Their Explanatory Power on Psychological Adjustment

**DOI:** 10.3389/fpsyg.2019.00630

**Published:** 2019-03-27

**Authors:** Jesus Montero-Marin, Maria C. Perez-Yus, Ausias Cebolla, Joaquim Soler, Marcelo Demarzo, Javier Garcia-Campayo

**Affiliations:** ^1^Primary Care Prevention and Health Promotion Research Network (RedIAPP), Zaragoza, Spain; ^2^Department of Psychology and Sociology, University of Zaragoza, Zaragoza, Spain; ^3^Department of Personality, Assessment and Psychological Treatments, University of Valencia, Valencia, Spain; ^4^CIBERObn Ciber Physiopathology of Obesity and Nutrition, Madrid, Spain; ^5^Hospital de la Santa Creu i Sant Pau, Institut d’Investigació Biomèdica Sant Pau–IIB Sant Pau, Universitat Autònoma de Barcelona, Barcelona, Spain; ^6^Centro de Investigación Biomédica en Red de Salud Mental, Barcelona, Spain; ^7^Mente Aberta – Brazilian Center for Mindfulness and Health Promotion, Department of Preventive Medicine, Universidade Federal de São Paulo, São Paulo, Brazil; ^8^Miguel Servet Hospital and University of Zaragoza, Zaragoza, Spain; ^9^Aragon Institute for Health Research (IIS Aragon), Zaragoza, Spain

**Keywords:** religious beliefs, prayer, focused attention, open monitoring, compassion meditation, psychological adjustment, practice variables

## Abstract

There has been increased interest in the relationships between religiosity, meditation practice and well-being, but there is lack of understanding as to how specific religious components and distinct meditation practices could influence different positive and negative psychological adjustment outcomes. The aim of this study was to assess the explanatory power of religious beliefs and the practice of prayer, focused attention (FA), open monitoring (OM), and compassion meditation (CM) on psychological adjustment, taking into consideration a number of practice-related variables such as session length, frequency of practice and lifetime practice. Psychological adjustment was assessed by means of happiness, positive affect, depression, negative affect, and emotional overproduction. A cross-sectional design was used, with a final sample comprising 210 Spanish participants who completed an online assessment protocol. Hierarchical regressions were performed, including age, sex and psychotropic medication use in the first step as possible confounders, with the addition of religious beliefs and the practice of prayer, FA, OM, and CM in the second step. FA session length was related to all psychological adjustment outcomes: happiness (Δ*R*^2^ = 0.09, *p* = 0.002; β = 0.25, *p* = 0.001), positive affect (Δ*R*^2^ = 0.09, *p* = 0.002; β = 0.18, *p* = 0.014), depression (ΔR^2^ = 0.07, *p* = 0.004; β = -0.27, *p* < 0.001), negative affect (Δ*R*^2^ = 0.08, *p* = 0.007; β = -0.27, *p* < 0.001) and emotional overproduction (Δ*R*^2^ = 0.07, *p* = 0.013; β = -0.23, *p* = 0.001). CM session length was related to positive affect (β = 0.18, *p* = 0.011). CM practice frequency was associated with happiness (Δ*R*^2^ = 0.06, *p* = 0.038; β = 0.16, *p* = 0.041). Lifetime practice of FA was related to happiness (Δ*R*^2^ = 0.08, *p* = 0.007; β = 0.21, *p* = 0.030) and OM to emotional overproduction (Δ*R*^2^ = 0.08, *p* = 0.037; β = -0.19, *p* = 0.047). Religious beliefs and prayer seemed to be less relevant than meditation practices such as FA, OM, and CM in explaining psychological adjustment. The distinct meditation practices might be differentially related to distinct psychological adjustment outcomes through different practice-related variables. However, research into other forms of institutional religiosity integrating social aspects of religion is required.

## Introduction

Religiosity has been associated with a variety of psychological well-being outcomes and seems to provide a protective function against mental illness (Koenig and Larson, 2001; Hebert et al., 2007; Candy et al., 2012; Balbuena et al., 2013; Bonelli and Koenig, 2013; Barton et al., 2013; Macilvaine et al., 2013; Pargament and Lomax, 2013; Gonçalves et al., 2015). It has also been associated with longevity, better indicators of quality of life, more effective coping strategies, greater optimism and self-esteem, and less anxiety, depression and autolytic or alcoholic behaviors (Cotton et al., 2006; Pikó and Kovács, 2009; Taheri Kharame et al., 2014; Unterrainer et al., 2014). However, there is some controversy regarding the relationship between religiosity and psychological well-being, with some studies pointing out that it would depend on how they are defined (Lindeman et al., 2012), and whether groups of non-believers are also considered (Zuckerman et al., 2016). In addition, there is little understanding of the precise mechanisms by which religiosity might influence mental health (Peres et al., 2017).

Religiosity has been implicated in predicting treatment response among patients with mental disorders (Kim et al., 2015), but it may have both costs and benefits (Rippentrop et al., 2005; Weber and Pargament, 2014). It has been established that not only positive health consequences flow from religious engagement, and thus the identification of distinct dimensions of religiosity would enable the search for both positive and negative health-related outcomes (Seeman et al., 2003). In fact, whether religiosity is beneficial, detrimental or neutral with regard to psychological adjustment is a question that has been examined repeatedly (Koenig and Larson, 2001; Lewis et al., 1997; Schaefer, 1997). One concern is that whenever religiosity and psychological adjustment have been analyzed to assess their common variation, little attention has been given to the nature of religion itself, which is multidimensional and might incorporate different aspects. For instance, Wood (2016) proposed that religious communities that impose great efforts and obligations offer more opportunities for the development of self-regulation abilities, favoring well-being as a longitudinal product. It has also been suggested that people might only benefit from a religious affiliation if they actively practice their religion, and that people with religious beliefs but who do not practice would not gain substantial benefits (Berthold and Ruch, 2014). Furthermore, it has been proposed that different forms of religiosity might be differentially related to distinct forms of psychological adjustment (Hackney and Sanders (2003).

Religiosity implies affiliation with a specific religion and its dogmas – i.e., beliefs, practices and rituals associated with the sacred – and usually includes rules governing behavior (Koenig, 2009). However, there are people who consider themselves to be spiritual but not religious, understanding spirituality in individualistic and secular terms (Koenig, 2009). Spirituality is a personal and informal adherence to transcendent meanings and beliefs that is relatively free of the rules associated with religion (Huguelet and Koenig, 2007). Although there is no agreement on a single definition of spirituality, it is considered in terms of religion, because there are many similarities between the two concepts (Zimbauer and Pargament, 2005), forming a multifactorial construct (Koenig, 2008). Thus, religiosity is made up of domains that are institutional, i.e., integrating social aspects; ideological, i.e., focused on beliefs; and devotional, i.e., with reference to individual practices such as prayer. Hackney and Sanders (2003) suggested that institutional forms of religiosity provide the least relevant aspects of religion in existential terms, and therefore would produce few effects on psychological adjustment, compared with ideology and, more particularly, devotion. Religious beliefs could benefit mental health by providing a sense of meaning and purpose (Koenig, 2009; Weber and Pargament, 2014). On the other hand, religious practices, such as prayer, are common coping behaviors among patients suffering from mental illness (Koenig, 2009), and they could play a role in the recovery process (Tepper et al., 2001; Unterrainer et al., 2014; Johnson, 2018). As can be seen, psychological adjustment can be defined in several ways: as an absence of negative outcomes (e.g., depression, etc.), as a matter of happiness (e.g., well-being, etc.), or as a self-actualization process (e.g., identity integration, etc.). Based on the self-determination theory (Deci and Ryan, 1985), Hackney and Sanders (2003) proposed that religious beliefs and practices, when they are driven by an internal motivation, and thus based on ideology and more especially devotion, would be associated with positive well-being outcomes such as happiness or self-actualization.

Psychological adjustment could be developed through religious contemplative practices such as meditation (Gelderloos et al., 1990). Meditation is “a family of attentional and emotional regulatory training regimes developed for various ends, including the cultivation of well-being and emotional balance” (Lutz et al., 2008). Benefits of meditation for mental health have been documented in clinical and non-clinical populations (Khoury et al., 2013; Demarzo et al., 2015). Although meditation encompasses a group of practices that share distinctive features, it is not easy to reduce meditation to a single procedure (Ospina et al., 2007; Sedlmeier et al., 2012). Currently, the most studied forms of meditation are focused attention (FA), open monitoring (OM), and compassion meditation (CM) (Lippelt et al., 2014; Cebolla et al., 2017). FA includes practices that aim to narrow attentional scope by the cultivation of concentration on a single event/object, such as breathing or a candle flame. OM permits attentional scope to be expanded by focusing attentive contemplation onto any experience that may arise (thoughts, emotions or perceptions), with no selecting, over-identifying or judging of any particular experience. CM focuses on cultivating pro-social and empathic behaviors by the recognition of and desire to relieve pain and suffering for oneself and others. FA and OM are attentional types of meditation – i.e., they train processes associated with the regulation of attention – whereas CM is a form of constructive meditation – i.e., it replaces maladaptive self-schema with more adaptive conceptions of the self (Lippelt et al., 2014; Dahl et al., 2015).

The potential regulatory function of FA, OM, and CM on attentional and emotional processes may have an impact on the brain and behavior, affecting psychological adjustment (Lutz et al., 2008). The type of effect produced by meditation would likely vary according to the type of meditation that is practiced, because different types of meditation practices display distinct psychological processes, which could differentially impact emotional experiences (Lutz et al., 2008; Goyal et al., 2014). Previous research has shown that FA, OM, and CM might have distinct effects on attention, conflict monitoring and creativity, with different neural structures and electroencephalographic patterns being activated (Lee et al., 2012; Tang and Posner, 2013; Lippelt et al., 2014). Another study found that both attentional and CM practices reduced self-reported stress reactivity in healthy participants, but only compassion routes lowered the physiological response of the HPA axis and cortisol (Engert et al., 2017). Data regarding how different meditation practices are associated with distinct psychological adjustment domains are scarce, but attentional meditation procedures, such as those included in meditative therapy programs, are generally thought to target the alleviation of negative emotions (Khoury et al., 2013; Gotink et al., 2015), while CM techniques, such as loving-kindness, seem to improve positive emotions (Zeng et al., 2015). Likewise, not only would the kind of religiosity component and meditation technique have relevance, but also the inclusion of certain practice-related variables such as session length, frequency of practice and lifetime practice, whose entire absence constitute a widespread methodological shortcoming of many studies (Lykins and Baer, 2009; Soler et al., 2014). One exception is the study by Fredrickson et al. (2017) in which length and frequency of meditative practice in general were related to positive well-being outcomes but were not associated with negative ones. Although Cebolla et al. (2017) did not consider psychological adjustment outcomes, they observed that length of FA practice was particularly associated with dispositional mindfulness.

There has been increased interest in the relationships between religiosity, meditation and well-being in recent years, but there is a lack of understanding as to how specific religious components and meditation practices could influence different psychological adjustment outcomes. In this context, the aim of this study was to assess the explanatory power of ideological (e.g., religious beliefs) and devotional (e.g., practice of prayer, FA, OM, and CM) forms of religiosity on psychological adjustment, measured through positive outcomes (e.g., happiness and positive affect) and negative outcomes (e.g., depression, negative affect, and emotional overproduction), giving consideration to the practice-related variables of session length, frequency of practice and lifetime practice. Sex and age were also considered as possible confounders because both have been associated with mood disorders, such as depression. In general, women are diagnosed with depression about twice as often as men (Salk et al., 2017), and depressive symptoms show increased values for older people (Hinz et al., 2014), although the relationships between depression and sex seems to be weaker with age (Patten et al., 2016). The reasons for this are not clear. Biological, psychological and sociological factors have been proposed to explain it, although strong empirical studies are still lacking. The use of psychotropic agents has also been associated with emotional and mental problems (Estancial Fernandes et al., 2018). Therefore, we decided to control for sex, age and use of psychotropic medication, to subtract their possible influence from the above-mentioned relationships that are the main intention of the present study (Seeman et al., 2003).

Owing to the incipient state of the literature, we did not establish strong hypotheses beyond the idea that the previously described forms of religiosity would contribute positively to psychological adjustment – and that they would therefore be positively related to positive outcomes and negatively associated with negative outcomes. However, we proposed the heuristic that different forms of religiosity and meditation practice would have different impacts on the distinct types of psychological adjustment, and that this would depend on session length, frequency of practice and lifetime experience, after controlling for the possible effects of sex, age and psychotropic medication use. In summary, we expected that: (1) the devotional aspects of religiosity (i.e., prayer and meditation) would be more important than the ideological components (i.e., belief status) in terms of explaining psychological adjustment (Hackney and Sanders, 2003); (2) the attentional procedures of meditation (i.e., FA and OM) would reduce negative outcomes, while CM would improve positive outcomes (Khoury et al., 2013; Gotink et al., 2015; Zeng et al., 2015); and (3) length of practice would have greater impact on psychological adjustment than the other practice-related variables (Cebolla et al., 2017).

## Materials and Methods

### Design, Procedure, and Participants

A cross-sectional study design was used in order to evaluate the explanatory power of religious beliefs and the practice of prayer and meditation over positive and negative psychological adjustment outcomes, through the practice-related variables of session length, frequency of practice and lifetime experience, after controlling for sex and age. Participants completed an assessment protocol in the Spanish language via a commercial online survey system^1^. A link to this protocol was posted on several Spanish websites related to religion, meditation and psychology (monasteries, meditation associations, scientific associations, etc.), as well as on non-professional social media (e.g., Facebook). A total of 599 subjects accessed the website, of whom 487 voluntarily agreed to participate and 365 completed the survey. The only inclusion criterion established was to answer all the practice-related variables of the survey in full. Given this condition, the final sample comprised 210 participants, of whom 62.9% were female, with a mean age of 43.11 years (11.04) and a range of 18–74 years. As regards education, 20.0% held a Ph.D. degree, 70.5% were university graduates, and 9.5% had only secondary studies; and therefore, the sample was composed mainly of participants with a high cultural level. With regard to health status, 87.1% did not suffer from any chronic disease, and 87.6% were not taking any psychotropic medication. In terms of religious adscription, 30.0% were affiliated as Christians, 13.3% as Buddhists, and 56.7% were non-believers. In summary, the participants were Spanish adults – mostly women, with a university degree, without chronic diseases and not taking any psychotropic medication – of whom a little under half had Christian or Buddhist religious beliefs, with the others being non-believers.

### Compliance With Ethical Standars

#### Ethics Statement

All procedures performed in studies involving human participants were in accordance with the ethical standards of the institutional and/or national research committee and with the 1964 Helsinki declaration and its later amendments or comparable ethical standards. The study protocol was approved by the local ethics committee - Ethics Committee of Aragon (PI12/00083) - and all participants had signed a written (online) consent form indicating their willingness to participate. They were informed about the purpose of the study, and it was made clear to them that their answers would be treated confidentially. In order to allow for replication studies, the data, and procedures of the present study are available in an open repository after anonymization.

### Measurements

#### Sociodemographic Information

We collected information on sex and age, not only to describe the sample but also to control for these possible confounding factors when analyzing the main relationships of interest, as mentioned previously. Other descriptive variables of the sample were education (primary, secondary, graduate, and postgraduate studies), presence of chronic disease (yes vs. no) and whether they were taking psychotropic medication (yes vs. no). We also asked about religious affiliation (Christian, Buddhist, others, none).

#### Religious Belief Status and Practice-Related Variables

-Religious belief status was assessed by only one item, as religiosity measures have consistently been estimated (Norenzayan and Hansen, 2006; Gebauer et al., 2016), with two possible options: “believer” vs. “non-believer.” Measures for the amount of prayer and meditation practice were also included to assess the specific practice-related variables (i.e., length of sessions in minutes; practice frequency: “daily,” “3–4 times/week,” “once a week or less,” “never”; and lifetime experience in months) for each type of practice independently (i.e., prayer, FA, OM, CM). We included a short description of each technique in order to ensure standardization among participants.

#### Psychological Adjustment Outcomes

-Pemberton Happiness Index (PHI): this scale measures happiness in the general population, and thus can be considered a positive psychological adjustment outcome. The PHI includes eleven items related to different domains of remembered well-being (general, hedonic, eudaimonic, and social well-being), each with an 11-point Likert scale from 0 (“totally disagree”) to 10 (“totally agree”), and 10 items related to experienced well-being (i.e., positive and negative emotional events that possibly happened the day before), with dichotomous response options (yes vs. no). The remembered well-being score is calculated with the mean score of the first 11 items, which may vary between 0 and 10. The 10 items for experienced well-being are converted into a single score between 0 (zero positive experiences and 5 negative experiences) and 10 (5 positive experiences and no negative experiences). The mean of the remembered and experienced scores produces a combined well-being index (total PHI) ranging between 0 and 10 and can be used to monitor changes in well-being, with adequate psychometric properties (Hervas and Vázquez, 2013). The internal consistency of the PHI total scale in the present study as measured by Cronbach’s alpha was α = 0.88.

-Remission from Depression Questionnaire (RDQ): the RDQ has 41 items with 7 subscales that capture a broad array of depression-related domains, and offers a negative psychological adjustment outcome total score by including symptoms of depression, other symptoms, positive mental health, coping ability, functioning, life satisfaction and a general sense of well-being. The items refer to the previous week and are rated on a 3-point Likert scale (scored between 0, “not at all or rarely true.” and 2, “often or almost always true”). The RDQ has demonstrated excellent psychometric properties (Zimmerman et al., 2013), allowing a broad point of view of depressed patients’ status to be gained that is consistent with a bio-psycho-social approach (Zimmerman et al., 2014). The internal consistence reliability value of the RDQ total scale, which ranges between 0 and 82, was α = 0.95 in the present study.

-The Positive and Negative Affect Schedule (PANAS): the PANAS (Watson et al., 1988) is a self-report questionnaire that measures positive and negative affectivity, and thus offers both positive and negative psychological adjustment outcomes. This instrument consists of a list of 20 adjectives, 10 per subscale (e.g., positive: “interested”; negative: “guilty”), which are rated on a 5-point Likert-type scale (between 1, “nothing,” and 5, “very much”), using the time instructions desired by the researcher. Present-moment instructions were used in this study. Each subscale ranges between 10 and 50. The PANAS has been validated in the Spanish language with good psychometric properties (Sandín et al., 1999). The internal consistency of the positive and negative affect scales in the present study was α = 0.90 and α = 0.88, respectively.

-Emotional Overproduction Scale (EOPS): this scale consists of 13 items that explore the role of emotional overproduction, conceptualized as the tendency to simultaneously experience negative emotions and feelings during sad episodes (Hervas and Vazquez, 2011), and thus can be considered as a negative psychological adjustment outcome. Emotional overproduction has been found to be associated with ruminative responses, which have a relevant role in the onset, duration and severity of depressive episodes. Participants are asked to rate how often they typically experience different negative emotions during sad episodes according to a 5-point Likert-type scale (between 1, “never,” and 5, “always”). The Spanish EOPS has proved to have good psychometric properties (Hervas and Vazquez, 2011). The internal consistence of the EOPS total scale, which ranges between 13 and 65, was α = 0.90 in the present study.

### Data Analysis

A descriptive analysis was first performed of religious belief status, prayer and meditation session length, frequency of practice and lifetime practice, as well as the psychological adjustment outcomes of PHI, RDQ, PANAS-Positive, PANAS-Negative and EOPS by using means and standard deviations (SD), or frequencies and percentages (%), according to the specific nature of each variable.

We subsequently compared the positive and negative psychological adjustment outcomes of PHI, RDQ, PANAS-Positive, PANAS-Negative and EOPS according to the belief status using *t*-tests and Cohen’s *d* effect size measures, and we also used Pearson’s r coefficients in order to bivariately evaluate the correlations of session length, frequency of practice and lifetime practice of prayer, FA, OM, and CM over the positive and negative psychological adjustment outcomes, without subtracting the possible influence of sex, age and the other variables of interest in order to give a general non-adjusted overview of the relationships proposed.

Finally, hierarchical multivariate linear regression models were built to assess the explanatory power of religious belief status, and session length, frequency of practice and lifetime practice of prayer, FA, OM and CM on the psychological adjustment outcomes of PHI, RDQ, PANAS-Positive, PANAS-Negative and EOPS. We used these analyses to estimate the impact of religious belief status and the practice of prayer and meditation techniques, considered in their entirety, on psychological adjustment, in addition to identifying the most important predictors. For this purpose, we built three regression models, one for each practice-related variable (session length, frequency of practice and lifetime practice) on each dependent variable (PHI, RDQ, PANAS-Positive, PANAS-Negative and EOPS), considering religious belief status and the corresponding prayer, FA, OM, and CM practice-related variables as independent factors. Specifically, the following predictors were included: age, sex and psychotropic medication use in the first step in order to control for possible variation patterns (Seeman et al., 2003; Estancial Fernandes et al., 2018), and religious belief status, and prayer, FA, OM, and CM, according to the different practice-related variables, in the second step. Standardized coefficients (β) were used to assess the individual contribution of the independent variables to explaining the psychological adjustment outcomes, and their statistical significance was established by the Wald test. Multiple determination coefficients (*R*^2^) were calculated to observe their grouped explanatory power in each regression model, and the statistical significance of the increment (Δ*R*^2^), obtained when going from the first hierarchical model to the next, was tested using ANOVA.

The Kolmogorov–Smirnov test was used to determine whether the conditional distribution of the residuals met the assumption of normality. It was confirmed that the Durbin–Watson (DW) values approached a value ≈2.00 to rule out autocorrelation problems in the errors, and that the variance inflation factor (VIF) and tolerance parameters (T) did not exceed critical values, in order to prevent multicollinearity problems in the regression models (Martínez-González, 2006).

All tests were bilateral, with a significance level of *p* < 0.05. There were no corrections for multiple comparisons given the highly exploratory nature of this study (Feise, 2002). The SPSS v19.0 statistical software package was used to perform the data analysis.

## Results

### Characteristics of Study Participants

Table 1 shows the general characteristics of study participants in terms of religious belief status, prayer and meditation practice-related variables, and psychological adjustment outcomes. As can be observed, 43.3% of participants assigned themselves as believers, and 56.7% as non-believers. The longest session length of practice was found in FA (Mn = 15.95 min; *SD* = 12.97), while the shortest session length of practice was observed in prayer (Mn = 8.57 min; *SD* = 17.52). Daily practice showed 34.3% of participants practicing FA; 27.1%, OM; 16.2%, prayer; and 8.6%, CM. The largest lifetime practice experience was found in FA (Mn = 82.75 months; *SD* = 102.69), while the shortest lifetime practice experience was observed in CM (Mn = 37.85 months; *SD* = 80.02). In general terms, and judging from the values obtained in each variable, the participants of the present study showed good psychological adjustment (Table 1).

**Table 1:** Descriptive data for religious belief status, practice-related variables, and psychological adjustment outcomes.

Variables	Total (*n* = 210)
Religious belief status, Yes	91 (43.3)
**Prayer**	
*-Session length (minutes)*	
Mn (*SD*)	8.57 (17.52)
*-Frequency of practice*	
Daily	34 (16.2)
3–4 times/week	16 (7.6)
Once a week or less	19 (9.0)
Never	141 (67.1)
*-Lifetime practice (months)*	
Mn (SD)	81.02 (161.78)
**Focused attention**	
*-Session length (minutes)*	
Mn (*SD*)	15.95 (12.97)
*-Frequency of practice*	
Daily	72 (34.3)
3–4 times/week	62 (29.5)
Once a week or less	50 (23.8)
Never	26 (12.4)
*-Lifetime practice (months)*	
Mn (*SD*)	82.75 (102.69)
**Open monitoring**	
*-Session length (minutes)*	
Mn (*SD*)	14.93 (15.81)
*-Frequency of practice*	
Daily	57 (27.1)
3–4 times/week	57 (27.1)
Once a week or less	58 (27.6)
Never	38 (18.1)
*-Lifetime practice (months)*	
Mn (*SD*)	72.28 (93.89)
**Compassion meditation**	
*-Session length (minutes)*	
Mn (*SD*)	12.91 (21.65)
*-Frequency of practice*	
Daily	18 (8.6)
3–4 times/week	36 (17.1)
Once a week or less	63 (30.0)
Never	93 (44.3)
*-Lifetime practice (months)*	
Mn (SD)	37.85 (80.02)
**PHI**, Mn (*SD*)	8.01 (1.11)
**PANAS-Positive**, Mn (*SD*)	37.86 (6.26)
**RDQ**, Mn (*SD*)	11.31 (12.33)
**PANAS-Negative**, Mn (*SD*)	15.28 (5.76)
**EOPS**, Mn (*SD*)	31.32 (9.09)

*PHI, Pemberton Happiness Index. PANAS-Positive, positive affect. RDQ, Remission from Depression Questionnaire. PANAS-Negative, negative affect. EOPS, Emotional Overproduction Scale Values are frequencies and percentages (%) except for age (in years), length of sessions (in minutes), experience of practice (in months), happiness (from 0 to 10), positive affect (from 10 to 50), depression (from 0 to 82), negative affect (from 10 to 50), and emotional overproduction (from 13 to 65), which are means (Mn) and standard deviations (SD)*.

### Religious Beliefs, Prayer, Meditation Practice, and Psychological Adjustment

There were no significant differences between believers and non-believers in the positive and negative psychological adjustment outcomes, although a certain trend was observed in the positive outcomes, favouring believers [(PHI: believers Mn = 8.18; *SD* = 1.01; non-believers Mn = 7.88; *SD* = 1.16; *d* = 0.28; *t* = 1.91; *p* = 0.058); (PANAS-Positive: believers Mn = 38.76; *SD* = 5.66; non-believers Mn = 37.17; *SD* = 6.61; *d* = 0.26; *t* = 1.83; *p* = 0.069); (RDQ: believers Mn = 10.80; *SD* = 11.47; non-believers Mn = 11.71; *SD* = 12.99; *d* = 0.07; *t* = 0.53; *p* = 0.600); (PANAS-Negative: believers Mn = 14.69; *SD* = 5.31; non-believers Mn = 15.73; *SD* = 6.06; *d* = 0.18; *t* = 1.30; *p* = 0.196); (EOPS: believers Mn = 30.68; *SD* = 10.29; non-believers Mn = 31.81; *SD* = 8.04; *d* = 0.12; *t* = 0.86; *p* = 0.392)]. Table 2 presents the raw correlations between the practice-related variables and the psychological outcomes. As can be seen, length of FA sessions was related to all the psychological outcomes (PHI: *r* = 0.20, *p* = 0.003; PANAS-Positive: *r* = 0.15, *p* = 0.026; RDQ: *r* = -0.21, *p* = 0.002; PANAS-Negative: *r* = -0.22, *p* = 0.002; EOPS: *r* = -0.18, *p* = 0.008). Length of CM sessions was related to PANAS-Positive (*r* = 0.21, *p* = 0.003). Practice frequency of prayer and CM was related to PHI (*r* = 0.16, *p* = 0.023; *r* = 0.16, *p* = 0.022; respectively). Lifetime practice of FA was related to all the outcomes (PHI: *r* = 0.23, *p* = 0.001; PANAS-Positive: *r* = 0.23, *p* = 0.001; RDQ: *r* = -0.18, *p* = 0.009; PANAS-Negative: *r* = -0.18, *p* = 0.011; EOPS: *r* = -0.19, *p* = 0.007). Lifetime practice of OM was related to all the outcomes (PHI: *r* = 0.23, *p* = 0.001; PANAS-Positive: *r* = 0.22, *p* = 0.001; RDQ: *r* = -0.17, *p* = 0.017; PANAS-Negative: *r* = -0.15, *p* = 0.029; EOPS: *r* = -0.24, *p* = 0.001). Lifetime practice of CM was related to PHI (*r* = 0.14; *p* = 0.047) and PANAS-Positive (*r* = 0.14; *p* = 0.046).

**Table 2:** Raw relationships between the practice-related variables of prayer and meditation techniques and the different psychological adjustment outcomes.

	Prayer		FA		OM		CM	
Length of
sessions	*r*	*p*	*r*	*p*	*r*	*p*	*r*	*p*
PHI	0.05	0.468	0.20	0.003	-0.05	0.503	0.13	0.054
PANAS-Positive	0.06	0.406	0.15	0.026	-0.04	0.607	0.21	0.003
RDQ	-0.07	0.350	-0.21	0.002	-0.03	0.612	-0.04	0.526
PANAS-Negative	-0.06	0.372	-0.22	0.002	-0.05	0.437	0.05	0.481
EOPS	0.01	0.881	-0.18	0.008	-0.04	0.578	-0.08	0.232
Practice frequency								
PHI	0.16	0.023	0.09	0.211	0.06	0.415	0.16	0.022
PANAS-Positive	0.10	0.176	-0.03	0.656	0.04	0.616	0.08	0.260
RDQ	-0.12	0.084	-0.13	0.055	-0.11	0.129	-0.09	0.203
PANAS-Negative	-0.13	0.052	-0.13	0.058	-0.08	0.270	-0.02	0.801
EOPS	-0.08	0.224	-0.03	0.665	-0.07	0.337	0.01	0.882
Lifetime practice								
PHI	0.10	0.159	0.23	0.001	0.23	0.001	0.14	0.047
PANAS-Positive	0.01	0.903	0.23	0.001	0.22	0.001	0.14	0.046
RDQ	-0.06	0.418	-0.18	0.009	-0.17	0.017	-0.09	0.180
PANAS-Negative	-0.06	0.389	-0.18	0.011	-0.15	0.029	-0.07	0.326
EOPS	-0.03	0.627	-0.19	0.007	-0.24	0.001	-0.08	0.251

*Prayer, practice of prayer. FA, focused attention. OM, open monitoring. CM, compassion meditation. PHI, Pemberton Happiness Index. PANAS-Positive, positive affect. RDQ, Remission from Depression Questionnaire. PANAS-Negative, negative affect. EOPS, Emotional Overproduction Scale. r, Pearson’s *r* coefficient. p, *p*-value*.

When carrying out the hierarchical regression analyses, separating out the practice-related variables, we observed that religious beliefs and length of sessions of prayer and meditation (Table 3) produced significant Δ*R*^2^ values from step 1 to step 2 in all the psychological adjustment outcomes: PHI (Δ*R*^2^ = 0.09; *p* = 0.002), PANAS-Positive (Δ*R*^2^ = 0.09; *p* = 0.002), RDQ (Δ*R*^2^ = 0.07; *p* = 0.004), PANAS-Negative (Δ*R*^2^ = 0.08; *p* = 0.007) and EOPS (Δ*R*^2^ = 0.07; *p* = 0.013). Specifically, FA session length contributed significantly to explaining PHI (β = 0.25; *p* = 0.001), PANAS-Positive (β = 0.18; *p* = 0.014), RDQ (β = -0.27; *p* < 0.001), PANAS-Negative (β = -0.27; *p* < 0.001) and EOPS (β = -0.23; *p* = 0.001). In addition, CM session length also contributed significantly to PANAS-Positive (β = 0.18; *p* = 0.011). On the other hand, religious beliefs and frequency of prayer and meditation practice (Table 3) produced significant Δ*R*^2^ values from step 1 to step 2 in PHI (Δ*R*^2^ = 0.06; *p* = 0.038), while only frequency of CM practice contributed to explaining PHI (β = 0.16; *p* = 0.041). Finally, religious beliefs and lifetime practice of prayer and meditation (Table 3) produced significant Δ*R*^2^ values from step 1 to step 2 in PHI (Δ*R*^2^ = 0.08; *p* = 0.007), PANAS-Positive (Δ*R*^2^ = 0.08; *p* = 0.007) and EOPS (Δ*R*^2^ = 0.08; *p* = 0.037). However, of these, only lifetime practice of FA contributed to explaining PHI (β = 0.21; *p* = 0.030), while lifetime practice of OM contributed to explaining EOPS (β = -0.19; *p* = 0.047). Age significantly and consistently explained (inversely) RDQ, PANAS-Negative and EOPS in steps 1 and 2. Sex was not consistently related to any of them. Psychotropic medication use was consistently related to RDQ and EOPS in steps 1 and 2.

**Table 3:** Hierarchical regression of (age, sex, medication) religious beliefs and practice-related variables of prayer and meditation on psychological adjustment.

				Sex		Sex		Medication		Beliefs		Prayer		FA		OM		CM	
Length of sessions		*R*^2^	ΔR^2^	β	*t*	β	*t*	β	*t*	β	*t*	β	*t*	β	*t*	β	*t*	β	*t*
PHI	Step 1	0.05*		0.11	1.55	0.13	1.76	-0.14	-1.97							
	Step 2	0.14***	0.09**	0.13	1.90	0.12	1.70	-0.14	-2.00*	0.08	1.09	0.03	0.47	0.25	3.53**	-0.12	-1.77	0.09	1.22
PANAS-Positive	Step 1	0.05*		0.16	2.30*	0.08	1.08	-0.12	-1.72							
	Step 2	0.14***	0.09**	0.19	2.74**	0.07	1.00	-0.11	-1.66	0.10	1.44	0.02	0.30	0.18	2.48*	-0.10	-1.42	0.18	2.58*
RDQ	Step 1	0.18***		-0.36	-5.36***	-0.06	-0.91	0.20	3.07**							
	Step 2	0.25***	0.07**	-0.37	-5.66***	-0.06	-0.90	0.21	3.23**	0.01	0.06	-0.03	-0.46	-0.27	-4.07***	0.02	0.34	-0.01	-0.16
PANAS-Negative	Step 1	0.06**		-0.19	-2.71**	-0.02	-0.29	0.14	1.92							
	Step 2	0.14**	0.08**	-0.19	-2.76**	-0.01	-0.14	0.15	2.19*	-0.06	-0.88	-0.03	-0.38	-0.27	-3.74***	0.01	0.12	0.10	1.44
EOPS	Step 1	0.09**		-0.21	-3.01**	-0.04	-0.60	0.20	2.85**							
	Step 2	0.16**	0.07*	-0.24	-3.42**	-0.04	-0.53	0.20	2.92**	-0.08	-1.08	0.07	0.91	-0.23	-3.24**	-0.01	-0.12	-0.04	-0.56
Practice frequency																
PHI	Step 1	0.05*		0.12	1.72	0.14	2.05*	-0.14	-2.09*							
	Step 2	0.11**	0.06*	0.10	1.44	0.12	1.72	-0.15	-2.20*	0.10	1.33	0.09	1.25	0.06	0.71	-0.02	-0.24	0.16	2.06*
PANAS-Positive	Step 1	0.04*		0.15	2.13*	0.07	1.05	-0.12	-1.75							
	Step 2	0.07	0.03	0.13	1.89	0.06	0.82	-0.13	-1.84	0.12	1.65	0.04	0.48	-0.07	-0.84	0.03	0.39	0.09	1.22
RDQ	Step 1	0.17***		-0.35	-5.28***	-0.06	-0.92	0.20	3.12**							
	Step 2	0.21***	0.04	-0.35	-5.23***	-0.06	-0.84	0.20	3.00**	<0.01	0.02	-0.09	-1.23	-0.14	-1.87	-0.04	-0.47	-0.01	-0.07
PANAS-Negative	Step 1	0.06*		-0.20	-2.91**	-0.03	-0.49	0.14	2.03*							
	Step 2	0.10*	0.04	-0.20	-2.86**	-0.02	-0.35	0.14	1.96	-0.05	-0.71	-0.10	-1.31	-0.14	-1.83	-0.03	-0.31	0.06	0.79
EOPS	Step 1	0.09**		-0.21	-3.07**	-0.03	-0.43	0.19	2.74**										
	Step 2	0.10**	0.02	-0.21	-2.96**	-0.03	-0.39	0.18	2.67**	-0.04	-0.52	-0.07	-1.00	-0.02	-0.19	-0.07	-0.89	0.04	0.57
Lifetime practice																
PHI	Step 1	0.05*		0.11	1.52	0.14	1.90	-0.14	-1.98							
	Step 2	0.13**	0.08**	0.07	1.01	0.14	1.95	-0.16	-2.26*	0.08	1.05	-0.01	-0.07	0.21	2.19*	0.11	1.10	-0.05	-0.53
PANAS-Positive	Step 1	0.03		0.14	1.95	0.06	0.81	-0.10	-1.37										
	Step 2	0.11**	0.08**	0.14	1.88	0.08	1.05	-0.11	-1.62	0.12	1.67	-0.13	-1.67	0.18	1.88	0.10	1.03	-0.01	-0.15
RDQ	Step 1	0.16***		-0.34	-5.10***	-0.05	-0.73	0.19	2.86**							
	Step 2	0.20***	0.04	-0.34	-4.90***	-0.06	-0.85	0.21	3.11**	-0.03	-0.40	0.08	1.06	-0.19	-2.12*	-0.03	-0.34	0.07	0.79
PANAS-Negative	Step 1	0.06*		-0.20	-2.78**	-0.02	-0.29	0.12	1.77							
	Step 2	0.10*	0.04	-0.19	-2.54*	-0.02	-0.25	0.15	2.06*	-0.09	-1.17	0.04	0.54	-0.20	-2.06*	-0.03	-0.35	0.08	0.93
EOPS	Step 1	0.07**		-0.20	-2.85**	-0.01	-0.19	0.17	2.46*							
	Step 2	0.13**	0.06*	-0.19	-2.66**	-0.03	-0.46	0.18	2.61*	-0.05	-0.67	0.07	0.87	-0.13	-1.37	-0.19	-2.00*	0.12	1.41

Medication, psychotropic medication use (no vs. yes); Beliefs, belief status (believer vs. non-believer); Prayer, practice of prayer. FA, practice of focused attention. OM, practice of open monitoring. CM, practice of compassion meditation. Step 1: age and sex as independent variables. Step 2: age, sex, beliefs, and *length of sessions* (minutes) or *practice frequency* (times per week) or *lifetime practice* (hours) of prayer, FA, OM, and CO., as independent variables. PHI, Pemberton Happiness Index. PANAS-Positive, positive affect. RDQ, Remission from Depression Questionnaire. PANAS-Negative, negative affect. EOPS, Emotional Overproduction Scale. *R*^*2*^, determination coefficient. Δ*R*^*2*^, increment of R^*2*^. β, standardized slope. *t*, *t*-test. ^∗^*p* < 0.05. **p < 0.01. ^∗∗∗^*p* < 0.001.

The statistics related to the adequacy of residuals and factors were appropriate in all the models and allowed interpretation of the regression analyses with guarantees.

## Discussion

To the best of our knowledge, this is the first study to assess the explanatory power of religiosity by means of religif prayer and different meditation techniques such as FA, OM, and CM on a number of positive and negative psychological adjustment outcomes. In our sample, we observed that almost half of the participants had a believer status, and the most practiced meditation technique was FA, while CM was the least used.

The main result of the present study suggests that greater psychological adjustment can be associated in general, regardless of sex, age, psychotropic medication use, religious belief status and practice of prayer with training in meditation techniques, in line with previous prospective research, showing that meditation leads to greater well-being. This finding is in agreement with a growing body of evidence supporting the general hypothesis that meditation practice may be linked to psychological and physiological health-related processes, and that commitment to religiosity – in a broad sense – might also include negative experiences (e.g., feelings of being punished by God, anger at God, religious doubts, religious passivity, conflicts with other members, etc.), as well as negative beliefs and forms of coping, misunderstandings and miscommunication, which could counterbalance other possible positive effects (Seeman et al., 2003; Weber and Pargament, 2014). A recent study found that certain religiosity dimensions, such as spiritual experiences, values, forgiveness and organizational religiousness, were associated with higher mental health status, while the religiosity dimension of meaning was related to somatic symptoms (Kioulos et al., 2015). We have observed in the present study that “believers” showed higher values in the positive psychological adjustment outcomes, although with a non-significant trend and moderately low effects, which remained non-significant in the subsequent multivariate analyses. This trend might achieve levels of significance in a larger sample, but it would need to be tested. Our results suggest that any interactions, if they exist, are relatively weak. In this sense, the most parsimonious explanation is that there was not a strong relationship. It has been said that while active participation in religious communities could have salutary effects, liminal positions such as “believing but not belonging” may be accompanied by a higher risk of mental health problems, even greater than for atheists, showing that the relationships between religiosity, secularity and mental health could be complex (Baker et al., 2018). One possible explanation for the results obtained in the present study in this regard might be that the psychological benefits of religiosity could be contingent on socio-cultural values, so that the benefits of religious adjustment would only be really achieved in scenarios of high country-level religiosity – i.e., societies with a high social value of religion, which is not the case of the modern Spanish society on which the present study was carried out (Gebauer et al., 2016).

There is insufficient knowledge regarding the mechanisms that mediate the effect of religion on mental health and well-being, although it has been proposed that some spiritual practices could facilitate awareness states that help to identify values such as patience, perseverance, kindness, compassion, forgiveness, gratitude, altruism, equanimity and wisdom, which could play an important role in this regard (Chaudhry, 2008; Baetz and Toews, 2009; Sharma and Singh, 2018). In the present study, the practice of meditation was related to psychological adjustment. It has been said that the relationship between meditation practice and values seems to be mediated by decentering (Franquesa et al., 2017), one of the main mechanisms of action in meditation, which allows one to become separated from one’s thoughts, enabling the person to observe experiences with greater clarity, and thus to choose more wisely in line with personal values (Shapiro et al., 2006; Kocovski et al., 2009; Garland et al., 2015). Decentering is to a great extent developed with mindful breathing, which is a type of FA technique, but it is not developed in the same way by other techniques such as loving-kindness meditation, which is designed to increase feelings of compassion (Feldman et al., 2010). This is in line with our results, because the length of FA sessions was consistently associated with positive and negative psychological adjustment variables, and this is interesting because only FA session length, but not OM or CM session length, has been related to observing, awareness, non-judging and non-reacting to inner experiences, which are the most important facets of mindfulness (Cebolla et al., 2017).

Mindfulness refers to an awareness that emerges by intentionally focusing attention on the present moment experience in a non-judgemental or non-evaluative way (Kabat-Zinn, 2005). The relationship between meditation practice and mindfulness has been clearly established (de Castro, 2015), as it has also been established between mindfulness and psychological health (Carmody et al., 2008; Khoury et al., 2013; Demarzo et al., 2015). Greeson et al. (2011) suggest that mindfulness partly mediates the association between increased spiritual experiences and improved mental health related to quality of life. In general, mindfulness has been associated with a number of cognitive functions (Lippelt et al., 2014), but there are few studies that deal with the effect of the different meditation techniques on psychological adjustment. In a study where three techniques (FA, body scan and yoga) were compared (Sauer-Zavala et al., 2012), the body scan technique was shown to be associated with reductions in rumination and describing, whereas FA was associated with an increase in non-judging of the inner experience. In another study (Carmody and Baer, 2008), FA was associated with improvements in most facets of mindfulness and several measures of symptoms and well-being, suggesting that the practice of FA meditation increases mindfulness abilities, which in turn lead to symptom reduction and improved well-being. Thus, the length of FA sessions – and perhaps also lifetime practice – could be related to psychological adjustment through the development of decentering and mindfulness skills, allowing reduced reactivity to repetitive thoughts and release from mental fixations by means of acceptance, which is an adaptive and fundamental component of third-generation psychotherapies (Öst, 2008; Feldman et al., 2010; Kliem et al., 2010; Athay, 2012; A-Tjak et al., 2015). Given that a high tendency for the mind to wander has been associated with lower levels of psychological well-being (Killingsworth and Gilbert, 2010), length of FA practice might be allowing the tendency for this wandering to be reduced, since attention is focused on activity in the present moment. However, owing to the limitations of the present study, we cannot rule out the assumption that people with a lower tendency for mind-wandering are able to practice FA for a longer period of time, and that, finally, these practitioners are the ones who acquire more experience in FA over time.

Nevertheless, it has also been suggested that type of meditation does not seem to be associated with level of mindfulness skills (Soler et al., 2014), and it is true that findings of specific effects of different meditation techniques are still inconsistent (Vettese et al., 2009). One study (O’Connor et al., 2015) found that religion-based practitioners had lower levels of guilt, empathic distress, depression and neuroticism, and higher levels of conscientiousness, resilience and altruism toward others, compared with secular meditators. When comparisons were made between techniques, findings suggested that those practicing contemplative prayer, a type of prayer that in fact could be considered in the attentional group of meditation practices (Dahl et al., 2015), showed higher levels of altruism toward strangers, and lower levels of neuroticism (O’Connor et al., 2015), with length of practice predicting positive outcomes, as we have found in FA meditation. We also found that the practice of prayer, in general terms, was associated with happiness in our study through bivariate analysis, but its individual contribution disappeared when using multivariate models. Previous pilot studies indicated that prayer, as well as meditative practice in general, might be related to a decreased sense of the self and could be an important factor in the relationship between religiosity and psychological well-being (Maltby et al., 1999; Johnstone, 2009). Therefore, we suggest that future research should study distinct techniques of prayer, and whether they are performed individually or in community, to elucidate possible factors of influence.

Within this prevailing discrepancy, we also found that CM session length contributed to explaining positive affect, and that frequency of CM practice was specifically related to happiness (lifetime practice of CM was also related to happiness and positive affect through bivariate analysis, but its explanatory power was null when using multivariate models). Interestingly, both positive affectivity and happiness are positive psychological adjustment outcomes, which suggest this type of meditation practice could specifically enhance this side of psychological adjustment, as we hypothesized. This could be possible through the processing of positive memories in an imagery-based way, which is a procedure habitually used in CM meditation (Nelis et al., 2015), and a central aspect of happiness when considering it as remembered well-being (Hervas and Vázquez, 2013). The “broaden-and-build theory of positive emotions” (Fredrickson, 2013) sustains that the human ability to experience pleasant emotions was selectively advantageous, and positive emotion experiences hold a value as resources in the face of life’s demands, extending momentary awareness in ways that build personal resources such as resilience, mental health and social integration (Fredrickson et al., 2017). Whether the practice of CM meditation genuinely contributes to pro-sociality beyond a simple desire to appear pro-social, considering that religiosity is strongly influenced by reputational concerns and stereotypes, is a question for future research (Galen, 2012).

On the other hand, lifetime practice of OM was negatively associated with emotional overproduction in the present study (lifetime practice of OM and also lifetime practice of FA were related to all the psychological outcomes through bivariate analysis, but their explanatory power disappeared when using multivariate models). Emotional overproduction, as the tendency to simultaneously experience an elevated number of negative emotions and feelings during sad episodes (Hervas and Vazquez, 2011), has been associated with rumination and difficulties in emotion regulation. OM is one of the main techniques of meditation and it is usually practiced by experienced meditators who have developed mindfulness mechanisms, such as emotion regulation, as a consequence of the practice (Hölzel et al., 2011), which is aligned with our results. OM could be expected to affect this tendency, perhaps by improving perceptual processes, and especially by decreasing psychological reactivity (Tang and Posner, 2013).

The main limitations of the present study were the cross-sectional design used, which did not allow us to establish causal relationships, and the non-random sampling procedure, which may have resulted in a selection bias (e.g., the high proportion of participants with university-level education), thus reducing the representativeness of our results. These have been two of the main limitations of research in the field of religiosity and spirituality to date (Seeman et al., 2003). It is important to highlight the great effort required in order to recruit regular meditators, and that the sample size we obtained allowed us to develop a statistical analysis with a relatively high power, enabling control for possible socio-demographic confounders such as age, sex, and psychotropic medication use. In this sense, we observed that sex was not a determinant in explaining psychological adjustment, and this is not the first time this kind of result has been obtained, probably due to the influence of other factors such as work-family trajectories (Stordal et al., 2001; Engels et al., 2019). However, age was directly related to positive affect, and inversely related to depression, negative affect and emotional overproduction, which is contrary to the findings of other studies (Hinz et al., 2014). Nevertheless, age has also shown inconsistent results as a potential risk factor, for instance, for depression (Blazer, 2000; Djernes, 2006), and we believe one determining factor for obtaining our results was the fact that our sample did not include very elderly participants, who usually form a part when studying age as a risk factor for depression (Glaesmer et al., 2011). Psychotropic medication use was found to be associated with depression and emotional overproduction, which is consistent with previous research (Estancial Fernandes et al., 2018). Although our design did not allow us to establish directional relationships between them in this specific population of religious and meditator practitioners, those participants with emotional difficulties could be presumed to be taking medication to relieve their symptoms.

Thus, future research should also consider the impact of older age groups and other possible factors that moderate these relationships, but also the possible biases and socially desirable responding of participants when using self-report measures. Religious subjects may be influenced when reporting data of religious practice, and they could under-report symptoms in order to project a socially desirable image of themselves, due to illusory mental health states as a denial mechanism of suffering (Gillings and Joseph, 1996; de Oliveira Maraldi, 2018). Another limitation was the method used to measure the practice-related variables of prayer and meditation techniques – by means of one single item for each, which were reported through a recall method – and whose test-retest reliability was not assessed. This issue is particularly complex when measuring the specific effects of distinct spiritual techniques, because most practitioners usually combine different types in their routines, and which is why the measure of distinct practice-related variables makes real sense. In addition, we assessed religiosity and spirituality by means of the presence of religious beliefs and prayer and meditation practices, but other forms of institutional religiosity integrating social aspects such as church attendance, reading of religious texts and other aspects of religious services seems mandatory because congregational support might also be related to health outcomes (Johnstone, 2009). In this sense, the context of practice could play a differential role, e.g., retreats at monasteries seems to be a good source of improvements (Shaku et al., 2013; Montero-Marin et al., 2016). It would be also necessary to consider that religious communities may differentially affect well-being depending on identity values, such as sexual orientation (Hamblin and Gross, 2013), and to differentiate “intrinsic” vs. “extrinsic” forms of religiosity – religion as an end in itself or as a means of obtaining personal/social benefits, or to distinguish between atheists and agnostics in the group of non-believers (Zuckerman et al., 2016). Finally, follow-ups would be interesting because the effects of religiosity and meditation on psychological adjustment variables may vary over time. Despite all of these limitations, this study has a number of strengths. For instance, the classification of meditation techniques was based on the most widely used methods, while also being based on traditional sources. The variables used to estimate psychological adjustment covered a wide range of outcomes. Moreover, to our knowledge, this is the first study to explore the relationships between religiosity and psychological adjustment, integrating the presence/absence of religious beliefs – from both Christian and Buddhist traditions – and prayer and meditation practice-related variables as possible sources of variation.

## Conclusion

Religious belief status was not significantly related to psychological adjustment in the context of our study, and prayer was only significantly related to happiness in the raw analysis. However, we found preliminary evidence that distinct meditation practices might be differentially related to distinct psychological adjustment outcomes, through different practice-related variables.

We observed that devotional aspects of religiosity were more important in order to explain psychological adjustment (hypothesis 1). Our study has pointed out that some meditation practices could fit better with some psychological adjustment variables, answering the question of which indicators of practice would predict them. This is in line with previous studies that demonstrated the differential efficacy of meditation practices in promoting mindfulness skills (Lykins and Baer, 2009; Soler et al., 2014; Cebolla et al., 2017), or even the distinct relationships between specific facets of mindfulness and psychological symptoms (Cash and Whittingham, 2010; Colgan et al., 2015). All of this is important in order to understand the relationships between meditation practice and psychological adjustment, and has relevant implications. For instance, we can make use of specific forms of practice, depending on the aspects of psychological adjustment we are interested in enhancing, through meditation practice. We have seen that FA session length might be related to the entire range of psychological adjustment outcomes, that length and frequency of CM practice could be related to positive psychological adjustment outcomes, and that lifetime practice of OM techniques may be potentially related to self-regulation processes of negative emotions. Thus, CM would contribute to explaining the positive aspects of psychological adjustment, and the attentional procedures of meditation (e.g., FA and OM) would reduce the negative outcomes, as we proposed (hypothesis 2). However, we have also seen that the attentional practice of FA might improve positive outcomes. This could especially be manifested by the length of sessions in that longer practice sessions would provide greater benefits, which is aligned with our initial assumption that length of practice would have higher impact than the other practice-related variables (hypothesis 3). However, the explanatory power of all these relationships, although significant, was not very large, meaning that these results are a starting point. More research is needed to investigate possible benefits of other specific forms of religiosity, such as institutionalized ones, in addition to other types of meditation practices and other possible interactions with the practice-related variables. Thus, new studies confirming these effects and their stability over time are required.

It has been suggested that religiosity can be approached for health providers inside healthcare systems in several ways, for instance, under a body-mind-spirit model based on an Eastern approach, with possible improvements in physical and mental health (Chan et al., 2001, 2006). A new proposal has currently been developed for going “back to the future” in Western medicine by means of religiosity (Cayley, 2015). Research and training in religiosity/spirituality practices directed toward psychologists and mental health providers, should address and include the findings of these kinds of studies in order to refine prescription through an evidence-based approach.

## Data Availability

In order to permit replication studies, data are available at the following open access repository: http://doi.org/10.3886/E108603V1.

## Author Contributions

JG-C, AC, MD, and JS contributed to the conception and design of the study. MP-Y organized the database. JM-M performed the statistical analysis, wrote the first draft of the manuscript and subsequent versions. All authors contributed to manuscript revision, and read and approved the submitted version.

## Conflict of Interest Statement

The authors declare that the research was conducted in the absence of any commercial or financial relationships that could be construed as a potential conflict of interest.

## Footnotes

1 www.surveymonkey.com

## References

[B1] Athay M. (2012). Satisfaction with life scale (SWLS) in caregivers of clinically-referred youth: psychometric properties and mediation analysis. *Adm. Policy. Ment. Health* 39 41–50. 10.1007/s10488-011-0390-8 22407554 PMC3578659

[B2] A-Tjak J. Davis M. Morina N. Powers M. Smits J. Emmelkamp P. (2015). A meta-analysis of the efficacy of acceptance and commitment therapy for clinically relevant mental and physical health problems. *Psychother. Psychosom.* 84 30–36. 10.1159/000365764 25547522

[B3] Baetz M. Toews J. (2009). Clinical implications of research on religion, spirituality, and mental health. *Can. J. Psychiatry* 54 292–301. 10.1177/070674370905400503 19497161

[B4] Baker J. O. Stroope S. Walker M. H. (2018). Secularity, religiosity, and health: physical and mental health differences between atheists, agnostics, and nonaffiliated theists compared to religiously affiliated individuals. *Soc. Sci. Res.* 75 44–57. 10.1016/j.ssresearch.2018.07.003 30080491

[B5] Balbuena L. Baetz M. Bowen R. (2013). Religious attendance, spirituality, and major depression in canada: a 14-year follow-up study. *Can. J. Psychiatry* 58 225–232. 10.1177/070674371305800408 23547646

[B6] Barton Y. A. Miller L. Wickramaratne P. Gameroff M. J. Weissman M. M. (2013). Religious attendance and social adjustment as protective against depression: a 10-year prospective study. *J. Affect. Disord.* 146 53–57. 10.1016/j.jad.2012.08.037 22959684 PMC3582716

[B7] Berthold A. Ruch W. (2014). Satisfaction with life and character strengths of non-religious and religious people: it’s practicing one’s religion that makes the difference. *Front. Psychol.* 14:876. 10.3389/fpsyg.2014.00876 25177303 PMC4132480

[B8] Blazer D. G. (2000). Psychiatry and the oldest old. *Am. J. Psychiatry* 157 1915–1924. 10.1176/appi.ajp.157.12.1915 11097951

[B9] Bonelli R. M. Koenig H. G. (2013). Mental disorders, religion and spirituality 1990 to 2010: a systematic evidence-based review. *J. Relig. Health* 52 657–673. 10.1007/s10943-013-9691-4 23420279

[B10] Candy B. Jones L. Varagunam M. Speck P. Tookman A. King M. (2012). Spiritual and religious interventions for well-being of adults in the terminal phase of disease. *Coch. Database. Syst. Rev.* 5:CD007544. 10.1002/14651858.CD007544.pub2 22592721 PMC13054467

[B11] Carmody J. Baer R. (2008). Relationships between mindfulness practice and levels of mindfulness, medical and psychological symptoms and well-being in a mindfulness-based stress reduction program. *J. Behav. Med.* 31 23–33. 10.1007/s10865-007-9130-7 17899351

[B12] Carmody J. Reed G. Kristeller J. Merriam P. (2008). Mindfulness, spirituality, and health-related symptoms. *J. Psychosom. Res.* 64 393–403. 10.1016/j.jpsychores.2007.06.015 18374738

[B13] Cash M. Whittingham K. (2010). What facets of mindfulness contribute to psychological well-being and depressive, anxious, and stress-related symptomatology? *Mindfulness* 1 177–182. 10.1007/s12671-010-0023-4

[B14] Cayley W. J. (2015). A proposal for getting “back to the future” of spirituality in medicine. *Acad. Med.* 90 546–547. 10.1097/ACM.0000000000000691 25919073

[B15] Cebolla A. Campos D. Galiana L. Oliver A. Tomás J. M. Feliu-Soler A. (2017). Exploring relations among mindfulness facets and various meditation practices: do they work in different ways? *Conscious. Cogn.* 49 172–180. 10.1016/j.concog.2017.01.012 28214767

[B16] Chan C. Ho P. Chow E. (2001). A body-mind-spirit model in health: an Eastern approach. *Soc. Work Health Care* 34 261–282. 10.1300/J010v34n03_02 12243428

[B17] Chan C. Ng S. Ho R. Chow A. (2006). East meets West: applying Eastern spirituality in clinical practice. *J. Clin. Nurs.* 15 822–832. 10.1111/j.1365-2702.2006.01649.x 16879375

[B18] Chaudhry H. R. (2008). Psychiatric care in Asia: spirituality and religious connotations. *Int. Rev. Psychiatry* 20 477–483. 10.1080/09540260802397602 19012134

[B19] Colgan D. D. Christopher M. Michael P. Wahbeh H. (2015). The body scan and mindful breathing among veterans with PTSD: type of intervention moderates the relationship between changes in mindfulness and post-treatment depression. *Mindfulness* 7 372–383. 10.1007/s12671-015-0453-0 PMC7451147 32863982

[B20] Cotton S. Puchalski C. Sherman S. Mrus J. Peterman A. Feinberg J. (2006). Spirituality and religion in patients with HIV/AIDS. *J. Gen. Intern. Med.* 24:994 10.1007/s11606-009-1052-3 PMC1924778 17083501

[B21] Dahl C. J. Lutz A. Davidson R. J. (2015). Reconstructing and deconstructing the self: cognitive mechanisms in meditation practice. *Trends Cogn. Sci.* 19 515–523. 10.1016/j.tics.2015.07.001 26231761 PMC4595910

[B22] de Castro J. (2015). Meditation has stronger relationships with mindfulness, kundalini, and mystical experiences than yoga or prayer. *Conscious. Cogn.* 35 115–127. 10.1016/j.concog.2015.04.022 26002763

[B23] de Oliveira Maraldi E. (2018). Response bias in research on religion, spirituality and mental health: a critical review of the literature and methodological recommendations. *J. Relig. Health* 10.1007/s10943-018-0639-6 [Epub ahead of print]. 29770899

[B24] Deci E. L. Ryan R. M. (1985). *Intrinsic Motivation and Self-Determination in Human Behavior.* New York, NY: Plenum Press 10.1007/978-1-4899-2271-7

[B25] Demarzo M. M. Montero-Marin J. Cuijpers P. Zabaleta-del-Olmo E. Mahtani K. R. Vellinga A. (2015). The efficacy of mindfulnessbased interventions in primary care: a meta-analytic review. *Ann. Fam. Med.* 13 573–582. 10.1370/afm.1863 26553897 PMC4639383

[B26] Djernes J. K. (2006). Prevalence and predictors of depression in populations of elderly: a review. *Acta Psychiatry Scand.* 113 372–387. 10.1111/j.1600-0447.2006.00770.x 16603029

[B27] Engels M. Weyers S. Moebus S. Jöckel K. H. Erbel R. Pesch B. (2019). Gendered work-family trajectories and depression at older age. *Aging Ment. Health* 10.1080/13607863.2018.1501665 [Epub ahead of print]. 30621439

[B28] Engert V. Kok B. E. Papassotiriou I. Chrousos G. P. Singer T. (2017). Specific reduction in cortisol stress reactivity after social but not attention-based mental training. *Sci. Adv.* 3:e1700495. 10.1126/sciadv.1700495 28983508 PMC5627978

[B29] Estancial Fernandes C. S. de Azevedo R. Goldbaum M. Barros M. (2018). Psychotropic use patterns: are there differences between men and women? *PLoS One* 13:e0207921. 10.1371/journal.pone.0207921 30475871 PMC6257918

[B30] Feise R. J. (2002). Do multiple outcome measures require p-value adjustment? *BMC Med. Res. Methodol.* 2:8. 10.1186/1471-2288-2-8 12069695 PMC117123

[B31] Feldman G. Greeson J. Senville J. (2010). Differential effects of mindful breathing, progressive muscle relaxation, and loving-kindness meditation on decentering and negative reactions to repetitive thoughts. *Behav. Res. Ther.* 48 1002–1011. 10.1016/j.brat.2010.06.006 20633873 PMC2932656

[B32] Franquesa A. Cebolla A. García-Campayo J. Demarzo M. Elices M. Pascual J. C. (2017). Meditation practice is associated with a values-oriented life: the mediating role of decentering and mindfulness. *Mindfulness* 8 1259–1268. 10.1007/s12671-017-0702-5

[B33] Fredrickson B. L. (2013). Positive emotions broaden and build. *Adv. Exp. Soc. Psychol.* 47 1–53. 10.1016/B978-0-12-407236-7.00001-2

[B34] Fredrickson B. L. Boulton A. J. Firestine A. M. Cappellen P. V. Algoe S. B. Brantley M. M. (2017). Positive emotions correlates of meditation practice: a comparison of mindfulness meditation and loving-kindness-meditation. *Mindfulness* 8 1623–1633. 10.1007/s12671-017-0735-9 29201247 PMC5704778

[B35] Galen L. W. (2012). Does religious belief promote prosociality? A critical examination. *Psychol. Rep.* 138 876–906. 10.1037/a0028251 22925142

[B36] Garland E. L. Farb N. A. Goldin P. Fredrickson B. L. (2015). Mindfulness broadens awareness and builds eudaimonic meaning: a process model of mindful positive emotion regulation. *Psychol. Inq.* 26 293–314. 10.1080/1047840X.2015.1064294 27087765 PMC4826727

[B37] Gebauer J. E. Sedikides C. Schönbrodt F. D. Bleidorn W. Rentfrow P. J. Potter J. (2016). The religiosity as social value hypothesis: a multi-method replication and extension across 65 countries and three levels of spatial aggregation. *J. Pers. Soc. Psychol.* 113 e18–e39. 10.1037/pspp0000104 27442765

[B38] Gelderloos P. Hermans H. J. Ahlscröm H. H. Jacoby R. (1990). Transcendence and psychological health: studies with long-term participants of the transcendental meditation and TM-Sidhi program. *J. Psychol.* 124 177–197. 10.1080/00223980.1990.10543215 2187979

[B39] Gillings V. Joseph S. (1996). Religiosity and social desirability: Impression management and selfdeceptive positivity. *Pers. Indiv. Diff.* 21 1047–1050. 10.1016/S0191-8869(96)00137-7

[B40] Glaesmer H. Riedel-Heller S. Braehler E. Spangenberg L. Luppa M. (2011). Age- and gender-specific prevalence and risk factors for depressive symptoms in the elderly: a population-based study. *Int. Psychogeriatr.* 23 1294–1300. 10.1017/S1041610211000780 21729425

[B41] Gonçalves J. P. Lucchetti G. Menezes P. R. Vallada H. (2015). Religious and spiritual interventions in mental health care. *Psychol. Med.* 45 2937–2949. 10.1017/S0033291715001166 26200715 PMC4595860

[B42] Gotink R. A. Chu P. Busschbach J. J. V. Benson H. Fricchione G. L. Hunink M. G. M. (2015). Standardised mindfulness-based interventions in healthcare: an overview of systematic reviews and meta-analyses of RCTs. *PLoS One* 10:e0124344. 10.1371/journal.pone.0124344 25881019 PMC4400080

[B43] Goyal M. Singh S. Sibinga E. Gould N. Rowland-Seymour A. Sharma R. (2014). *Meditation Programs for Psychological Stress and Well-Being.* Rockville, MD: Agency for Healthcare Research and Quality. 24501780

[B44] Greeson J. M. Webber D. M. Smoski M. Brantley J. G. Ekblad A. G. Suarez E. C. (2011). Changes in spirituality partly explain health-related quality of life outcomes after mindfulness-based stress reduction. *J. Behav. Med.* 34 508–518. 10.1007/s10865-011-9332-x 21360283 PMC3151546

[B45] Hackney C. H. Sanders G. S. (2003). Religiosity and mental health: a meta-analysis of recent studies. *J. Sci. Study Relig.* 42 43–55. 10.1111/1468-5906.t01-1-00160

[B46] Hamblin R. Gross A. M. (2013). Role of religious attendance and identity conflict in psychological well-being. *J. Relig. Health* 52 817–827. 10.1007/s10943-011-9514-4 21761275

[B47] Hebert R. Dang Q. Schulz R. (2007). Religious beliefs and practices are associated with better mental health in family caregivers of patients with dementia: findings from the REACH study. *Am. J. Geriatr. Psychiatry* 15 292–300. 10.1097/01.JGP.0000247160.11769.ab 17158632

[B48] Hervas G. Vazquez C. (2011). What else do you feel when you feel sad? Emotional overproduction, neuroticism and rumination. *Emotion* 11 881–895. 10.1037/a0021770 21517169

[B49] Hervas G. Vázquez C. (2013). Construction and validation of a measure of integrative well-being in seven languages: the pemberton happiness index. *Health Qual. Life Outcomes* 11:66. 10.1186/1477-7525-11-66 23607679 PMC3637073

[B50] Hinz A. Finck C. Gómez Y. Daig I. Glaesmer H. Singer S. (2014). Anxiety and depression in the general population in Colombia: reference values of the hospital anxiety and depression scale (HADS). *Soc. Psychiatry Psychiatr. Epidemiol.* 49 41–49. 10.1007/s00127-013-0714-y 23748887

[B51] Hölzel B. K. Lazar S. W. Gard T. Schuman-Olivier Z. Ott U. (2011). How does mindfulness meditation work? proposing mechanisms of action from a conceptual and neural perspective. *Pers. Psychol. Sci.* 6:537. 10.1177/1745691611419671 26168376

[B52] Huguelet P. Koenig H. G. (2007). *Religion and Spirituality in Psychiatry.* New York, NY: Cambridge University Press.

[B53] Johnson K. A. (2018). Prayer: a helpful aid in recovery from depression. *J. Relig. Health* 57 2290–2300. 10.1007/s10943-018-0564-8 29383592

[B54] Johnstone B. (2009). Spirituality, religion and health outcomes research: findings from the center on religion and the professions. *Mo. Med.* 106 141–144. 19397115

[B55] Kabat-Zinn J. (2005). *Full Catastrophe Living: Using the Wisdom of Your Body and Mind to Face Stress, Pain, and Illness.* New York, NY: Delta Trade Paperback/Bantam Dell.

[B56] Khoury B. Lecomte T. Fortin G. Masse M. Therien P. Bouchard V. (2013). Mindfulness-based therapy: a comprehensive meta-analysis. *Clin. Psychol. Rev.* 33 763–771. 10.1016/j.cpr.2013.05.005 23796855

[B57] Killingsworth M. Gilbert D. A. (2010). Wandering mind is an unhappy mind. *Science* 330:932. 10.1126/science.1192439 21071660

[B58] Kim N. Huh H. Chae J. (2015). Effects of religiosity and spirituality on the treatment response in patients with depressive disorders. *Compr. Psychiatry* 60 26–34. 10.1016/j.comppsych.2015.04.009 25956752

[B59] Kioulos K. T. Bergiannaki J. D. Glaros A. Vassiliadou M. Alexandri Z. Papadimitriou G. (2015). Religiosity dimensions and subjective health status in Greek students. *Psychiatriki* 26 38–44. 25880382

[B60] Kliem S. Kröger C. Kosfelder J. (2010). Dialectical behavior therapy for borderline personality disorder: a meta-analysis using mixed-effects modeling. *J. Consult. Clin. Psychol.* 78:936. 10.1037/a0021015 21114345

[B61] Kocovski N. L. Segal Z. V. Battista S. R. (2009). “Mindfulness and Psychopathology: Problem Formulation,” in *Clinical Handbook of Mindfulness*, ed. Didonna F. (New York, NY: Springer), 85–98. 10.1007/978-0-387-09593-6_6

[B62] Koenig H. G. (2008). Concerns about measuring “spirituality” in research. *J. Nerv. Ment. Dis.* 196 349–355. 10.1097/NMD.0b013e31816ff796 18477877

[B63] Koenig H. G. (2009). Research on religion, spirituality, and mental health: a review. *Can. J. Psychiatry* 54 283–291. 10.1177/070674370905400502 19497160

[B64] Koenig H. G. Larson D. B. (2001). Religion and mental health: evidence for an association. *Int. Rev. Psychiatry* 13 67–78. 10.1080/09540260124661

[B65] Lee T. M. C. Leung M. K. Hou W. K. Tang J. C. Y. Yin J. So K. (2012). Distinct neural activity associated with focused-attention meditation and loving-kindness meditation. *PLoS One* 7:e40054. 10.1371/journal.pone.0040054 22905090 PMC3419705

[B66] Lewis C. A. Lanigan C. Joseph S. de Fockert J. (1997). Religiosity and happiness: no evidence for an association among undergraduates. *Pers. Indiv. Diff.* 22 119–121. 10.1016/S0191-8869(97)88910-6 19928598

[B67] Lindeman M. Blomqvist S. Takada M. (2012). Distinguishing spirituality from other constructs: not a matter of well-being but of belief in supernatural spirits. *J. Nerv. Ment. Dis.* 200 167–173. 10.1097/NMD.0b013e3182439719 22297316

[B68] Lippelt D. Hommel B. Colzato L. (2014). Focused attention, open monitoring and loving kindness meditation: effects on attention, conflict monitoring, and creativity -a review. *Front. Psychol.* 23:1083. 10.3389/fpsyg.2014.01083 25295025 PMC4171985

[B69] Lutz A. Slagter H. A. Dunne J. D. Davidson R. J. (2008). Attention regulation and monitoring in meditation. *Trends Cogn. Sci.* 12 163–169. 10.1016/j.tics.2008.01.005 18329323 PMC2693206

[B70] Lykins E. L. B. Baer R. A. (2009). Psychological functioning in a sample of long-term practitioners of mindfulness meditation. *J. Cogn. Psychother.* 23 226–241. 10.1891/0889-8391.23.3.226

[B71] Macilvaine W. Nelson L. Stewart J. Stewart W. (2013). Association of strength of religious adherence to quality of life measures. *Compl. Ther. Clin. Pract.* 19 251–255. 10.1016/j.ctcp.2013.05.001 24199983

[B72] Maltby J. Lewis C. A. Day L. (1999). Religious orientation and psychological well-being: the role of the frequency of personal prayer. *Br. J. Health Psychol.* 4 363–378. 10.1007/s10943-011-9514-4 21761275

[B73] Martínez-González M. (2006). *Bioestadística Amigable*, 5th Edn. Madrid: Díaz de Santos.

[B74] Montero-Marin J. Puebla-Guedea M. Herrera-Mercadal P. Cebolla A. Soler J. Demarzo M. (2016). Psychological effects of a 1-month meditation retreat on experienced meditators: the role of non-attachment. *Front. Psychol.* 7:1935. 10.3389/fpsyg.2016.01935 28018270 PMC5149565

[B75] Nelis S. Holmes E. Palmieri R. Bellelli G. Raes F. (2015). Thinking back about a positive event: the impact of processing style on positive affect. *Front. Psychiatry* 6:3. 10.3389/fpsyt.2015.00003 25806003 PMC4353183

[B76] Norenzayan A. Hansen I. G. (2006). Belief in supernatural agents in the face of death. *Pers. Soc. Psychol. Bull.* 32 174–187. 10.1177/0146167205280251 16382080

[B77] O’Connor L. E. O. Rangan R. K. Berry J. W. Stiver D. J. Hanson R. Ark W. (2015). Empathy, compassionate altruism and psychological well-being in contemplative practitioners across five traditions. *Psychology* 6 989–1000. 10.4236/psych.2015.68096

[B78] Ospina M. B. Bond K. Karkhaneh M. Tjosvold L. Vandermeer B. Liang Y. (2007). Meditation practices for health: state of the research. *Evid. Rep. Technol. Assess.* 155 1–263. PMC4780968 17764203

[B79] Öst L. (2008). Efficacy of the third wave of behavioral therapies: a systematic review and meta-analysis. *Behav. Res. Ther.* 46 296–321. 10.1016/j.brat.2007.12.005 18258216

[B80] Pargament K. Lomax J. (2013). Understanding and addressing religion among people with mental illness. *World Psychiatry* 12 26–32. 10.1002/wps.20005 23471791 PMC3619169

[B81] Patten S. B. Williams J. V. Lavorato D. H. Wang J. L. Bulloch A. G. Sajobi T. (2016). The association between major depression prevalence and sex becomes weaker with age. *Soc. Psychiatry Psychiatr. Epidemiol.* 51 203–210. 10.1007/s00127-015-1166-3 26743882

[B82] Peres M. F. P. Kamei H. H. Tobo P. R. Lucchetti G. (2017). Mechanisms behind religiosity and spirituality’s effect on mental health, quality of life and well-being. *J. Relig. Health* 57 1842–1855. 10.1007/s10943-017-0400-6 28444608

[B83] Pikó B. Kovács E. (2009). Is religiosity a protective factor? social epidemiologic study of adolescent psychological health. *Orv. Hetil.* 150 1903–1908. 10.1556/OH.2009.28704 19801357

[B84] Rippentrop E. Altmaier E. Chen J. Found E. Keffala V. (2005). The relationships between religion/spirituality and physical health, mental health, and pain in a chronic pain population. *Pain* 116 311–321. 10.1016/j.pain.2005.05.008 15979795

[B85] Salk R. H. Hyde J. S. Abramson L. Y. (2017). Gender differences in depression in representative national samples: meta-analyses of diagnoses and symptoms. *Psychol. Bull.* 143 783–822. 10.1037/bul0000102 28447828 PMC5532074

[B86] Sandín B. Chorot P. Lostao L. Joiner T. Santed M. Valiente R. (1999). Escala PANAS de afecto positivo y negativo: validación factorial y convergencia transcultural. *Psicothema* 11 37–51.

[B87] Sauer-Zavala S. Walsh E. Eisenlohr-Moul T. Lykins E. (2012). Comparing mindfulness-based intervention strategies: differential effects of sitting meditation, body scan, and mindful yoga. *Mindfulness* 4 383–388. 10.1007/s12671-012-0139-9

[B88] Schaefer W. E. (1997). Religiosity, spirituality, and personal distress among college students. *J. Coll. Stud. Dev.* 38 633–644.

[B89] Sedlmeier P. Eberth J. Schwarz M. Zimmermann D. Haarig F. Jaeger S. (2012). The psychological effects of meditation: a meta-analysis. *Psychol. Bull.* 138 1139–1171. 10.1037/a0028168 22582738

[B90] Seeman T. Dubin L. Seeman M. (2003). Religiosity/spirituality and health. A critical review of the evidence for biological pathways. *Am. Psychol.* 58 53–63. 10.1037/0003-066X.58.1.53 12674818

[B91] Shaku F. Tsutsumi M. Goto H. Saint Arnoult D. (2013). Measuring the effects of zen training on quality of life and mental health among Japanese monk trainees: a cross-sectional study. *J. Altern. Complement. Med.* 20 406–410. 10.1089/acm.2013.0209 24266527

[B92] Shapiro S. L. Carlson L. E. Astin J. A. Freedman B. (2006). Mechanisms of mindfulness. *J. Clin. Psychol.* 62 373–386. 10.1002/jclp.20237 16385481

[B93] Sharma S. Singh K. (2018). Religion and well-being: the mediating role of positive virtues. *J. Relig. Health* 58 119–131. 10.1007/s10943-018-0559-5 29353383

[B94] Soler J. Cebolla A. Feliu-Soler A. Demarzo M. Pascual J. Baños R. (2014). Relationship between meditative practice and self-reported mindfulness: the MINDSENS composite index. *PLoS One* 9:e86622. 10.1371/journal.pone.0086622 24466175 PMC3899282

[B95] Stordal E. Bjartveit Krüger M. Dahl N. H. Krüger Ø Mykletun A. Dahl A. A. (2001). Depression in relation to age and gender in the general population: the nord-trøndelag health study (HUNT). *Acta Psychiatr. Scand.* 104 210–216. 10.1034/j.1600-0447.2001.00130.x 11531658

[B96] Taheri Kharame Z. Zamanian H. Foroozanfar S. Afsahi S. (2014). Religious wellbeing as a predictor for quality of life in iranian hemodialysis patients. *Glob. J. Health Sci.* 6 261–269. 10.5539/gjhs.v6n4p261 24999150 PMC4825247

[B97] Tang Y. Posner M. (2013). Special issue on mindfulness neuroscience. *Soc. Cogn. Affect. Neur.* 8 1–3. 10.1093/scan/nss104 22956677 PMC3541496

[B98] Tepper L. Rogers S. A. Coleman E. M. Malony H. N. (2001). The prevalence of religious coping among persons with persistent mental illness. *Psychiatr. Serv.* 52 660–665. 10.1176/appi.ps.52.5.660 11331802

[B99] Unterrainer H. Lewis A. Fink A. (2014). Religious/spiritual Well-being, personality and mental health: a review of results and conceptual issues. *J. Relig. Health* 53 382–392. 10.1007/s10943-012-9642-5 22965652

[B100] Vettese L. Toneatto T. Stea J. Nguyen L. Wang J. (2009). Do mindfulness meditation participants do their homework? And does it make a difference? A review of empirical evidence. *J. Cogn. Psychother.* 23 198–224. 10.1891/0889-8391.23.3.198

[B101] Watson D. Clark L. Tellegen A. (1988). Development and validation of brief measures of positive and negative affect: the PANAS scales. *J. Pers. Soc. Psychol.* 54 1063–1070. 10.1037/0022-3514.54.6.1063 3397865

[B102] Weber S. Pargament K. (2014). The role of religion and spirituality in mental health. *Curr. Opin. Psychiatry* 27 358–363. 10.1097/YCO.0000000000000080 25046080

[B103] Wood C. (2016). Ritual well-being: toward a social signalling model of religion and mental health. *Relig. Brain Behav.* 7 223–243. 10.1080/2153599X.2016.1156556

[B104] Zeng X. Chiu C. P. K. Wang R. Oei T. P. S. Leung F. Y. K. (2015). The effect of loving-kindness meditation on positive emotions: a meta-analytic review. *Front. Psychol.* 6:1693. 10.3389/fpsyg.2015.01693 26579061 PMC4630307

[B105] Zimbauer B. J. Pargament K. I. (2005). “Religiousness and spirituality,” in *Handbook of the Psychology of Religion and Spirituality*, ed. Paloutzian R. F. (New York, NY: The Guilford Press).

[B106] Zimmerman M. Martinez J. Attiullah N. Friedman M. Toba C. Boerescu D. (2013). A new type of scale for determining remission from depression: the remission from depression questionnaire. *J. Psychiatr. Res.* 47 78–82. 10.1016/j.jpsychires.2012.09.006 23102820

[B107] Zimmerman M. Martinez J. H. Attiullah N. Friedman M. Toba C. Boerescu D. A. (2014). The remission from depression questionnaire as an outcome measure in the treatment of depression. *Depress. Anx.* 31 533–538. 10.1002/da.22178 24115164

[B108] Zuckerman P. Galen L. W. Pasquale F. L. (2016). *The Nonreligious: Understanding Secular People and Societies.* Oxford: Oxford University Press 10.1093/acprof:oso/9780199924950.001.0001

